# Study on the Grain Growth Behavior of Ultra-High Strength Stainless Steel

**DOI:** 10.3390/ma18051064

**Published:** 2025-02-27

**Authors:** Xiaohui Wang, Zhenbao Liu, Jiahao Chen, Jianxiong Liang, Zhiyong Yang, Wenyu Zhao, Shuai Tian

**Affiliations:** Central Iron and Steel Research Institute Co., Ltd., Beijing 100081, Chinayangzhiyong@nercast.com (Z.Y.); tianshuai@nercast.com (S.T.)

**Keywords:** ultra-high-strength stainless steel, austenite grain growth, mathematical model

## Abstract

In this work, we aimed to study the austenite grain growth behavior of an ultra-high-strength stainless steel within the temperature range of 900–1150 °C and holding time range of 0–120 min, using a metallographic microscope and metallographic image analysis software to perform a statistical analysis of grain size variation. The undissolved phases of the steel were investigated using a field emission scanning electron microscope (SEM) and transmission electron microscope (TEM). Within the temperature range of 900–950 °C, the grain growth rate of the steel was slow, while within the range of 1000–1150 °C, the grain growth rate was relatively fast. This is attributed to the precipitation of a large number of M_6_C-type carbides during the forging and annealing processes. In the temperature range of 900–950 °C, the solid solubility of the M_6_C phase was low and the pinning effect was significant, which hindered the growth of austenite grains. Above 950 °C, the carbides were dissolved extensively, weakening the pinning effect on the grain boundaries and accelerating the grain growth rate. A predictive mathematical model for the growth of the original austenite grains was established based on the Arrhenius equation, elucidating the effects of heating temperature, holding time, initial grain size, and number of carbides on the growth of austenite grains, providing a theoretical basis for heat treatment process design in actual production.

## 1. Introduction

High-strength stainless steel is one of the metal structural materials that has the best strength, toughness, and corrosion resistance; therefore, it is widely used in aerospace, aviation, marine, energy, and other technological fields [1,2,3]. In recent years, the development of such steels has reached a new level in terms of alloy composition design, smelting processes, and strength grades. Today, due to the adoption of advanced design concepts such as high strength, toughness, long service life, fatigue resistance, and environmental corrosion resistance in key load-bearing components of aircraft, the advantages of these steels have been fully demonstrated in various application fields. These characteristics have made them the focus of in-depth research by scholars both domestically and internationally [2,3]. Since the development of high-strength stainless steel in the 1940s, the first generation of semi-austenitic precipitation-hardened stainless steels, such as Stainless W, 17-4PH [4], 17-7PH [5], and PH15-7Mo, as well as the second generation of martensitic precipitation-hardened stainless steels, such as 15-5PH [6,7], PH13-8Mo [8], and Custom465 [9], have been developed. In 2008, the United States led the development of Ferrium^®^S53, a second-hardening ultra-high-strength stainless steel, which features an ultra-high strength of 1900 MPa and good toughness, becoming a representative of the third generation of high-strength stainless steels. Future aircraft designs will require high maneuverability and flexibility, and from the perspectives of spatial constraints and structural weight reduction, their materials will be expected to have higher strength, stiffness, and fracture toughness.

Grain size is one of the key material parameters that simultaneously affects the strength, plasticity, and toughness of steel [10,11,12]. When the heating temperature exceeds the austenitizing temperature of steel, the grain growth behavior of the original austenite grains changes with the heating temperature, holding time, alloy elements, and precipitates. For example, Sha and Sun [13] studied the grain growth behavior of Nb-V-Ti microalloyed steel and found that as the holding temperature increased, the austenite grain size gradually increased. In the temperature range below 1250 °C, titanium-rich carbonitrides were present at the austenite grain boundaries. Yu and Sun [14] studied 0.015% Nb steel and found that the critical grain growth temperature for austenite was 1240 °C, and in the temperature range of 1150–1230 °C, the austenite grain size of 0.015% Nb steel was smaller than that of steel without Nb. Research on 16MnNi4 low-alloy high-strength steel [15] found that when holding the temperature at 1200 °C for 5–30 min or at 1150 °C for 60 min, the austenite grain growth of the steel was rapid. In a study by Bao et al. [16] on the austenite grain growth behavior of medium manganese martensitic NM500 steel, when the heating temperature was below 950 °C, large amounts of undissolved nanoscale spherical and short rod-shaped V(C, N) particles were present in the experimental steel, which could effectively pin the austenite grain boundaries and slow down the grain growth. However, when the heating temperature exceeded 950 °C, a large number of V(C, N) particles were dissolved, leading to significant grain coarsening. Guo et al. [17] studied the austenite grain growth behavior of a 60Mn3Al3Ni2CrVNb quenching-type low-density steel and found that as the heating temperature increased, the austenite grain size gradually increased. Below 1100 °C, the grain growth was slow, while above 1100 °C, the grains coarsened significantly, with the coarsening temperature being around 1100 °C. In addition, a significant grain coarsening phenomenon occurred at 1250 °C. A microscopic analysis showed that the carbonitride of niobium was the key factor affecting the austenite grain growth behavior of the experimental steel. Yan et al. [18] studied the evolution of the microstructure of 00Cr21Ni6Mn9N stainless steel in the temperature range of 950–1200 °C and found that 00Cr21Ni6Mn9N steel without a Z phase could obtain uniform grain sizes above 1000 °C, while the steel containing a Z phase required temperatures above 1100 °C to achieve uniform grain sizes. Zheng et al. [19] studied the austenite grain growth law in high-nitrogen stainless steel in the temperature range of 950–1200 °C and found that the fine nitrides in the experimental steel inhibited the coarsening of the austenite grains due to the pinning effect. In practice, the growth behavior of primary austenite grains is affected by many factors, such as the heating temperature, holding time, initial grain size, and number of carbides.

This study investigated the austenite grain growth behavior of a 1900 MPa ultra-high-strength stainless steel. Due to its high alloy content, high deformation resistance, and other factors, traditional forging processes often use multi-stage heating with gradual temperature reduction to ensure effective grain refinement. During the forging and subsequent annealing processes, a large number of precipitates are formed or dynamically precipitated in the steel. These precipitates are usually coarse and distributed at the martensitic multi-level interfaces in the steel, which is detrimental to the steel’s plasticity and toughness. Therefore, solid solution treatment is required to eliminate coarse precipitates. If an appropriate solid solution treatment process is not adopted, then the grain structure will undergo abnormal coarsening, losing the effect of grain refinement during the forging process. At present, there are few studies on the grain growth behavior of ultra-high-strength stainless steel. This study explored austenite grain growth behavior in the steel, considering the effects of heating temperature, holding time, initial grain size, and number of carbides and not only providing useful insights for controlling the grain size but also offering a theoretical basis for the design of heat treatment processes in the actual production of this type of steel.

## 2. Experiments

**Figure 1:**
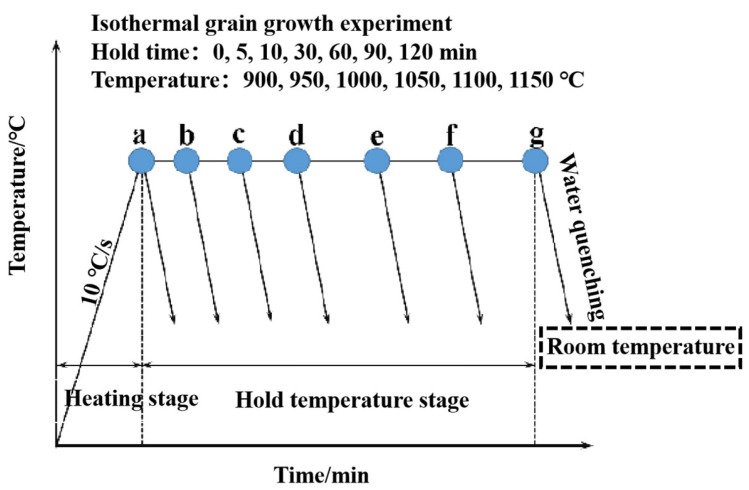
Schematic diagram of the austenite grain growth experimental process.

The experimental steel was prepared using vacuum induction and vacuum consumable remelting, with its main chemical composition being Fe-(0.08~0.15) wt.% C-(12.00~13.00) wt.% Cr-(2.00~3.00) wt.% Ni-(12.00~14.00) wt.% Co-(4.00~5.00) wt.% Mo-1.00 wt.% (W+V). Before forging, the steel ingot was heated to 1200 °C, homogenized, and then forged into billets 70 × 70 mm in size. Afterward, the billets were annealed at 680 °C for 24 h. Samples 10 × 10 × 2 mm in size were cut from the annealed billets to study the grain growth behavior of the high-strength stainless steel. The heat treatment experiments were carried out in a box-type resistance furnace, and the experimental process is shown in Figure 1. The samples were heated at a rate of 10 °C/s to the following temperatures: 900 °C, 950 °C, 1000 °C, 1050 °C, 1100 °C, and 1150 °C. After reaching the set temperature, the temperature was held for different holding times: 0 min, 5 min, 10 min, 30 min, 60 min, 90 min, and 120 min. The samples were then water-quenched to room temperature.

After the grain size samples were polished, they were etched at room temperature for 24 h in a solution of potassium permanganate and sulfuric acid (1 g KMnO_4_ + 10 mL H_2_SO_4_ + 90 mL H_2_O). The observation, acquisition, and analysis of images were performed using a ZEISS-40MAT metallographic microscope (OM, Carl Zeiss AG, Oberkochen, Germany) and domestic SISCIAS V8.0 metallographic image analysis software. The martensitic structure and undissolved phases of the steel were observed and analyzed using a JSM-6710F field emission scanning electron microscope (SEM, Jeol, Tokyo, Japan) and an H-800 transmission electron microscope (TEM, Hitachi, Tokyo, Japan). For the SEM analysis, the samples were polished and then etched in a copper chloride–hydrochloric acid solution (20 mL HCl + 10 g CuCl_2_ + 200 mL C_2_H_5_OH). For the TEM analysis, the samples were first mechanically polished to 40 μm and then thinned via double-spraying in a 5% HClO_4_-C_2_H_5_OH solution at a test temperature of −10 °C.

## 3. Results and Analysis

### 3.1. The Influence of Holding Temperature and Time on the Original Austenite Grains

**Figure 2:**
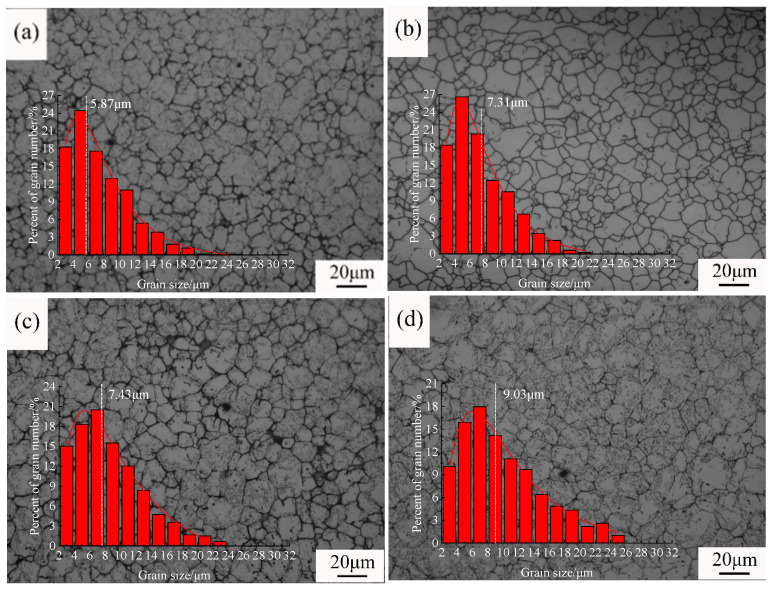

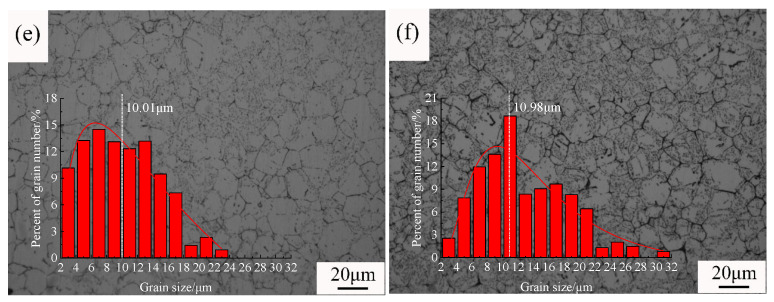
Morphology and grain size statistics of the original austenite grains after the temperature was held at 950 °C for different durations: (**a**) 0 min, (**b**) 5 min, (**c**) 10 min, (**d**) 30 min, (**e**) 60 min, and (**f**) 90 min. (There is no obvious change in the size of the original austenite grains).

**Figure 3:**
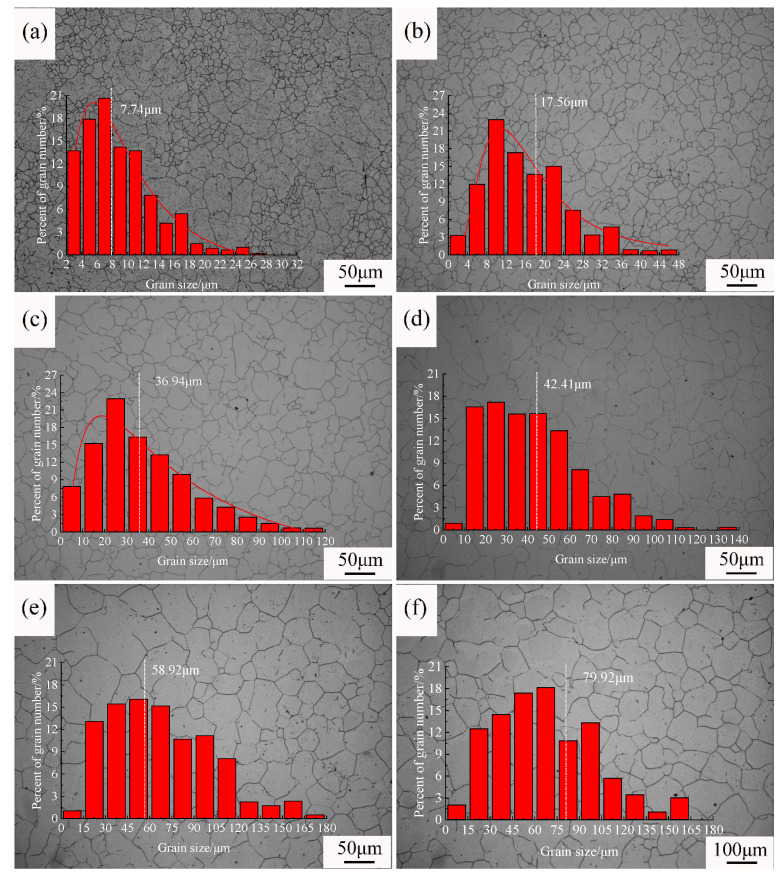
Morphology and grain size statistics of the original austenite grains after the temperature was held at 1050 °C for different durations: (**a**) 0 min, (**b**) 5 min, (**c**) 10 min, (**d**) 30 min, (**e**) 60 min, and (**f**) 90 min. (As the holding time increased, the mixed grain structure disappeared, and the grains began to coarsen).

**Figure 4:**
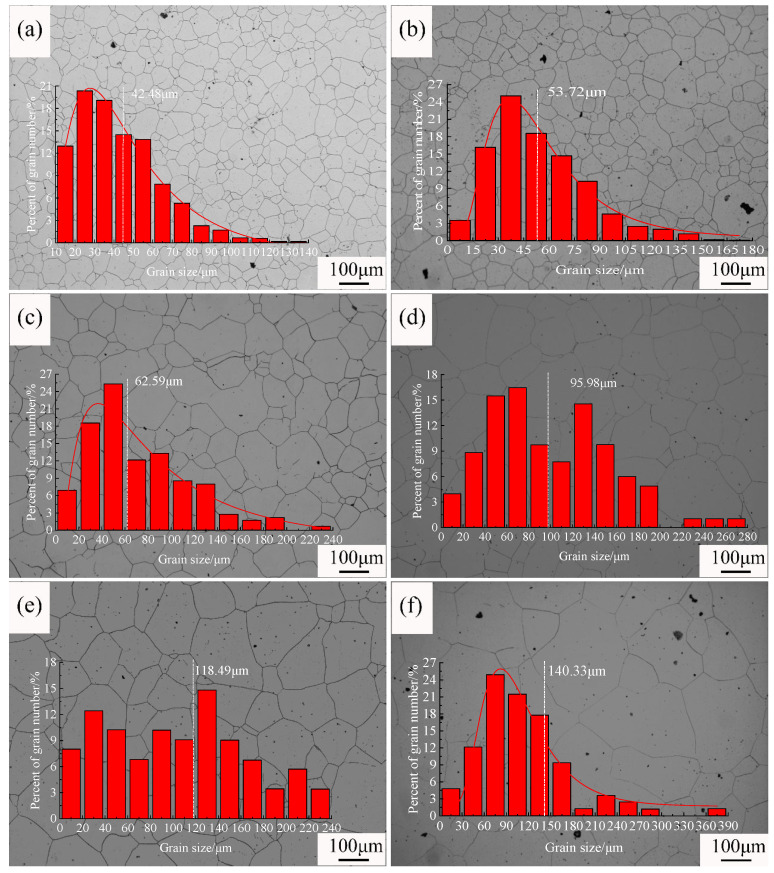
Morphology and grain size statistics of the original austenite grains after the temperature was held at 1150 °C for different durations: (**a**) 0 min, (**b**) 5 min, (**c**) 10 min, (**d**) 30 min, (**e**) 60 min, and (**f**) 90 min. (The grains began to grow significantly after the holding time started, and the size was significantly coarsened).

To investigate the growth behavior of original austenite grains during the heating of steel, different holding times were applied within the temperature range of 950–1150 °C. The typical grain structures resulting from different processes are shown in Figure 2, Figure 3 and Figure 4. Figure 2 shows the original austenite grain structures in the steel after the temperature was held at 950 °C for different durations. When the holding time was 0 min (Figure 2a), the original austenite grains in the steel were small equiaxed grains with an average grain size of 5.9 μm. Extending the holding time did not significantly change the size of the original austenite grains (Figure 2b–f). When the holding time was 90 min, the average grain size was 11.0 μm. Figure 3 shows the original austenite grain structures in the steel after the temperature was held at 1050 °C for different durations. When the holding time started, the grain structure in the steel was a bi-modal grain size distribution (Figure 3a,b), with an average grain size of 7.7 μm, comparable to the initial size at 950 °C. As the holding time increased, the bi-modal grain size distribution phenomenon disappeared, and the grains began to coarsen. When the holding time was 90 min (Figure 3f), the size of the original austenite grains was 79.9 μm. Figure 4 shows the original austenite grain structures in the steel after the temperature was held at 1150 °C for different durations. The grains began to grow significantly after the holding time started (Figure 4a), with an average grain size of 42.5 μm, increasing to seven times the initial size at 950 °C. When the holding time was 90 min, the grains further coarsened and the size of the original austenite grains in the steel reached 140.3 μm (Figure 4f). 

**Figure 5:**
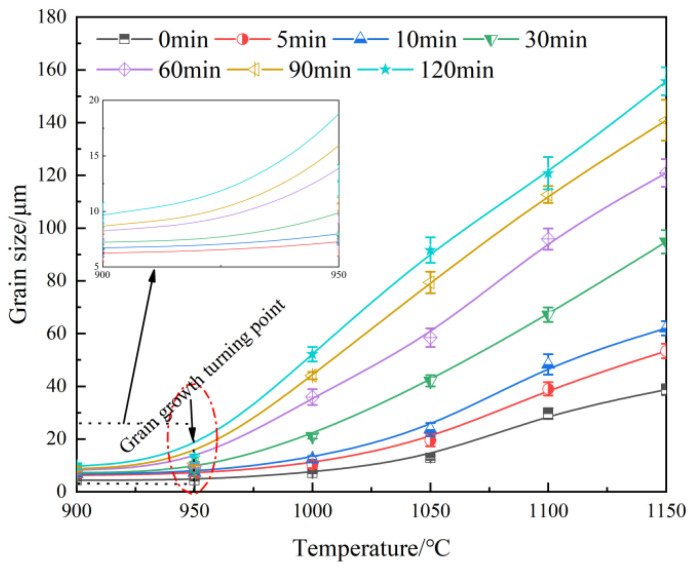
The evolution of the original austenite grain size with heating temperature at different holding times.

**Figure 6:**
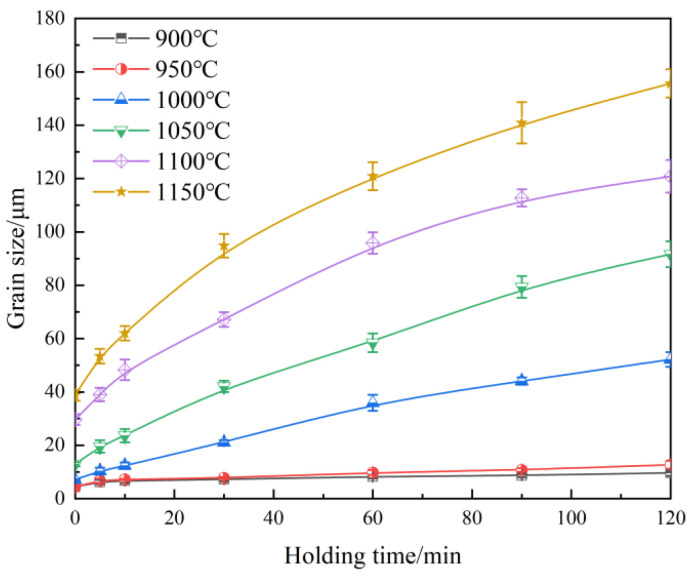
The evolution of the original austenite grain size with holding time at different holding temperatures.

The average size of the original austenite grains after different holding processes was statistically analyzed, and their evolution in the experimental steel with different austenitizing temperatures and holding times is shown in Figure 5 and Figure 6. The austenite grain size shows an exponential increase with temperature and a parabolic increase with holding time. When the temperature is within the range of 900–950 °C, the austenite grain size does not change significantly with longer holding times. However, when the temperature range is between 1000 °C and 1150 °C, the austenite grain size increases rapidly with longer holding times, and the grain growth rate decreases after the holding time exceeds 60 min. In the actual heat treatment process design, the appropriate heating temperature and holding time are selected according to the trend of the original grain growth (Figure 5 and Figure 6), which can effectively control the bi-modal grain size distribution phenomenon and improve the grain uniformity. At the same time, the abnormal grain growth is avoided, and the ideal grain size is obtained.

### 3.2. The Influence of Holding Temperature and Time on the Second Phase

**Figure 7:**
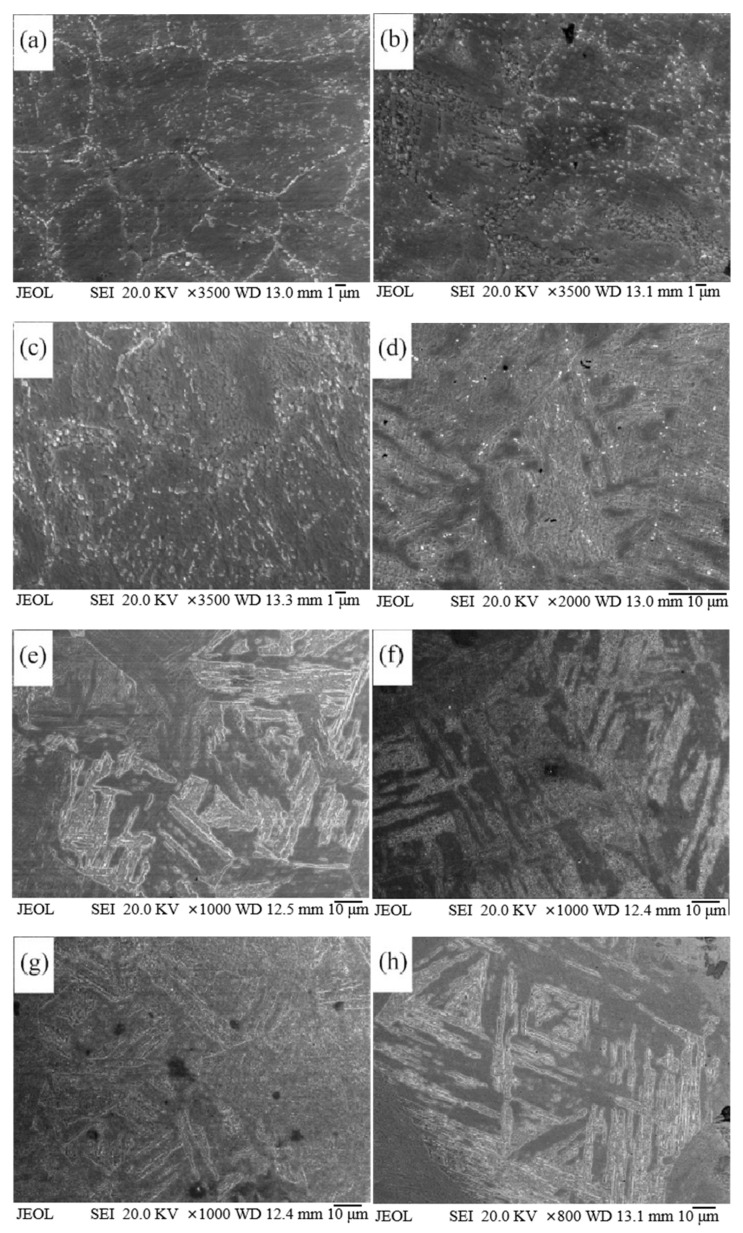
Microstructure of steel after different heating temperatures and holding times: (**a**) 950 °C for 5 min, (**b**) 950 °C for 60 min, (**c**) 950 °C for 120 min, (**d**) 1050 °C for 10 min, (**e**) 1050 °C for 60 min, (**f**) 1050 °C for 90 min, (**g**) 1150 °C for 5 min, and (**h**) 1150 °C for 15 min.

**Figure 8:**
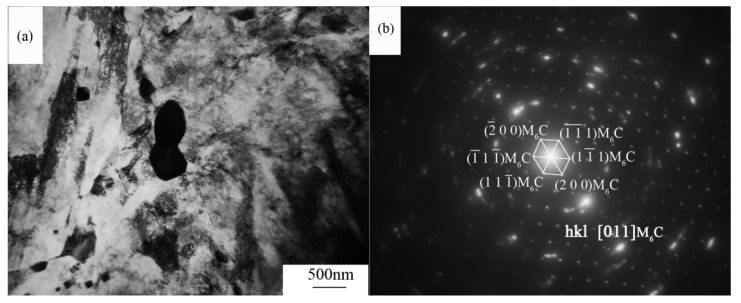
TEM micrographs and selected-area electron diffraction patterns of larger M_6_C carbides. (**a**) bright-field image, (**b**) selected-area electron diffraction patterns.

Figure 7 shows the microstructure of steel in different heat treatment conditions, with Figure 7a showing the microstructure of steel after annealing. In this Figure, a large amount of the second phase can be seen in the steel, which precipitates extensively during forging and annealing [20,21,22], mainly distributed at the original austenite grain boundaries and martensite multi-level organization interfaces. Figure 7a–h show the microstructure of steel after the temperature is held at 950 °C to 1150 °C for different durations. When the holding temperature is 950 °C, there is a large amount of the second phase at the original austenite grain boundaries. A TEM characterization analysis of the second phase in the steel after the temperature is held at 950 °C for 60 min is shown in Figure 8. The micro-characterization results show that the precipitated phase consists of M_6_C-type carbides. Due to the pinning effect of carbides, the grain structure does not grow significantly with a longer holding time. As the temperature increases, the grain structure of the steel gradually increases. When the holding temperature is 1050 °C, precipitates are only present at the original austenite grain boundaries when the holding time is 10 min. As the holding time is extended, the carbides gradually dissolve, and the grain structure grows significantly. When the holding temperature is 1150 °C, no large carbide particles are seen in the steel, indicating that the carbides have essentially dissolved into the matrix during the heating process. In the absence of the pinning effect of carbides, the grain structure coarsens significantly with a longer holding time. 

## 4. Model Establishment and Discussion

Holding time and temperature are two key factors in the growth of original austenite grains. According to the results in Figure 5 and Figure 6, 950 °C is the turning point temperature in the growth of austenite grains. Below 950 °C, the grain growth is slow, and above 950 °C, the grain growth rate increases rapidly. Therefore, the simulation and analysis of austenite grain growth need to be carried out in two temperature ranges: 900–950 °C and 1000–1150 °C. Thus, the growth model of austenite grains in the experimental steel was established in these two temperature ranges [23,24,25,26]:

(1) $$d_{t}^{n}-d_{0}^{n}=At^{m}exp(-\frac{Q}{RT})$$

where *d_t_* is the average grain size after the holding time *t*; *d*_0_ is the initial austenite grain size; *n* and *m* are the grain growth exponent and time exponent, respectively; *n* reflects the non-linear growth relationship between grain size and temperature and time; *m* reflects the influence of holding time on grain diameter; *t* is the holding time; *T* is the temperature; *R* is the gas constant; *Q* is the grain growth activation energy, which represents the apparent activation energy during grain growth, which reflects the energy required for grain boundary migration during grain growth; and *A* is a constant.

Taking the natural logarithm of both sides, Equation (1) can be deduced as follows:

(2) $$\ln\left(d_{t}^{n}-d_{0}^{n}\right)=lnA+mlnt-\frac{Q}{RT}$$

Assuming *n* to be 0.5, 1.0, 1.5, 2.0, 2.5, and 3.0, the corresponding *m*, *Q*, and *A* values can be obtained for different *n* values:

When holding temperature *T* is a constant value, *m* can be deduced as follows:

(3) $$m={\left.\frac{\partial [ln(d_{t}^{n}-d_{0}^{n})]}{\partial (lnt)}\right|}_{T=\mathrm{constant}}$$

When holding time *t* is a constant value, *Q* can be deduced as follows:

(4) $$Q={\left.\frac{-R\partial [ln(d_{t}^{n}-d_{0}^{n})]}{\partial (1/T)}\right|}_{t=\mathrm{constant}}$$

**Figure 9:**
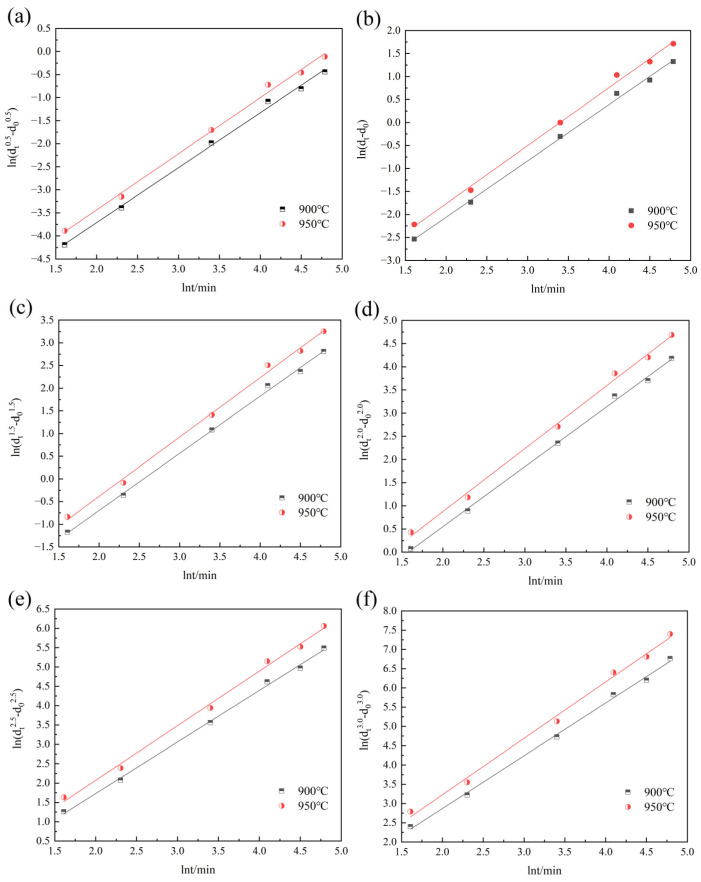
The relationship curves of $\ln\;(d_{t}^{n}-d_{0}^{n})$ and lnt at holding temperatures of 900–950 °C: (**a**) n = 0.5, (**b**) n = 1.0, (**c**) n = 1.5, (**d**) n = 2.0, (**e**) n = 2.5, and (**f**) n = 3.0.

**Figure 10:**
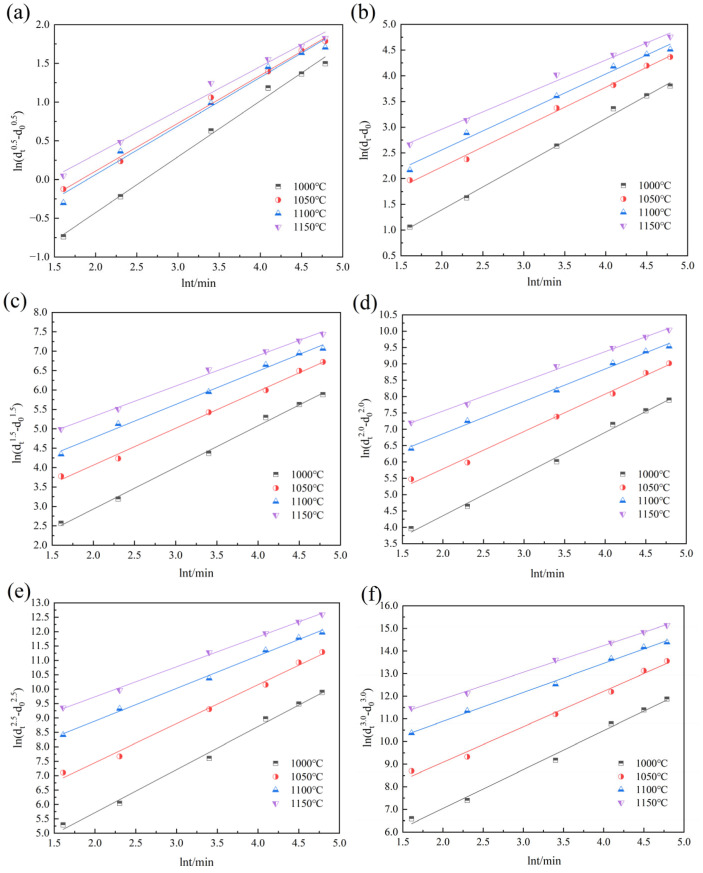
The relationship curves of $\ln\;(d_{t}^{n}-d_{0}^{n})$ and lnt at holding temperatures of 1000–1150 °C: (**a**) n = 0.5, (**b**) n = 1.0, (**c**) n = 1.5, (**d**) n = 2.0, (**e**) n = 2.5, and (**f**) n = 3.0.

**Figure 11:**
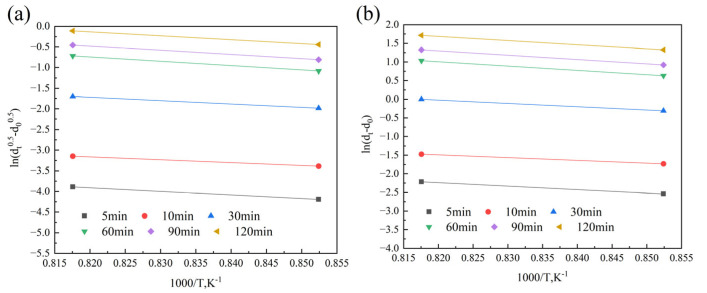

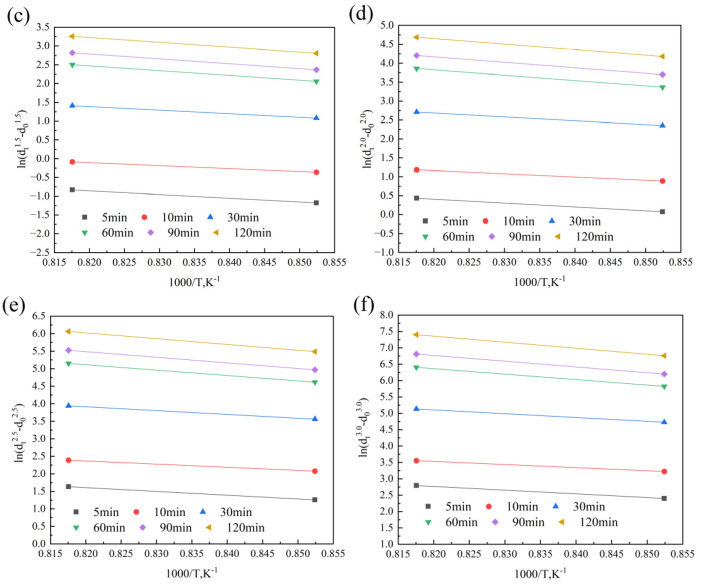
The relationship curves of $\ln\;(d_{t}^{n}-d_{0}^{n})$ and 1/T at holding temperatures of 900–950 °C: (**a**) n = 0.5, (**b**) n = 1.0, (**c**) n = 1.5, (**d**) n = 2.0, (**e**) n = 2.5, and (**f**) n = 3.0.

**Figure 12:**
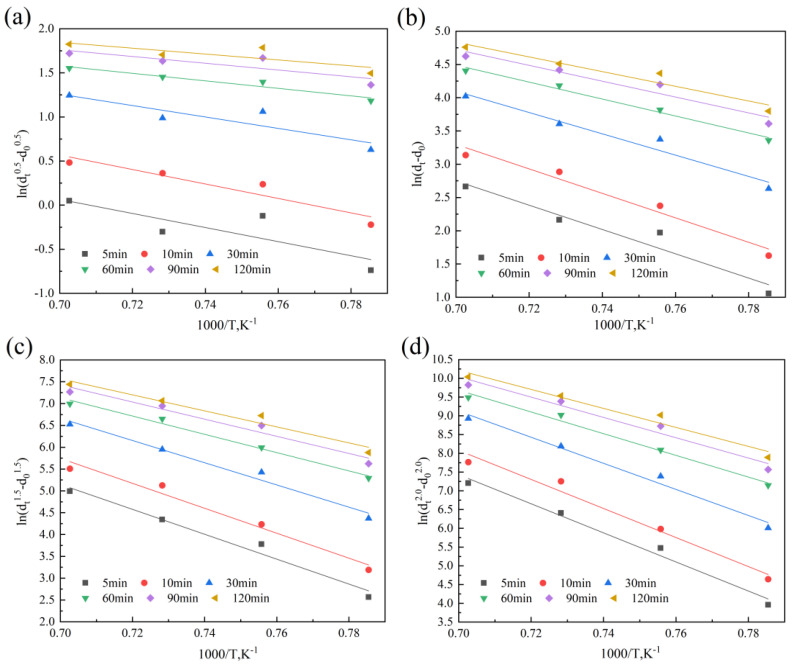

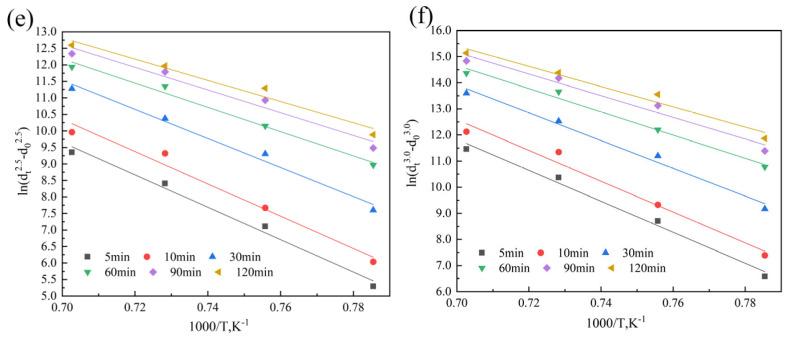
The relationship curves of $\ln\;(d_{t}^{n}-d_{0}^{n})$ and 1/T at holding temperatures of 1000–1150 °C: (**a**) n = 0.5, (**b**) n = 1.0, (**c**) n = 1.5, (**d**) n = 2.0, (**e**) n = 2.5, and (**f**) n = 3.0.

According to Equations (3) and (4), the values of *m* and *Q* corresponding to different *n* values can be obtained, as shown in Figure 9, Figure 10, Figure 11 and Figure 12. Figure 9 and Figure 10 show the relationship curves of $\ln\;(d_{t}^{n}-d_{0}^{n})$ and ln*t* in the 900–950 °C and 1000–1150 °C ranges, respectively. Figure 11 and Figure 12 show the relationship curves of $\ln\;(d_{t}^{n}-d_{0}^{n})$ and 1/*T* in the 900–950 °C and 1000–1150 °C ranges, respectively. From these curves, the corresponding *A* values and the original austenite grain growth equations based on different *n* values can finally be obtained.

To determine the optimal *n* value, an error equation is introduced [27]:

(5) $$f_{\mathrm{Error}(n)}=\sum_{i}^{n}{\left(d_{i}-d\right)}^{2}$$

where *d_i_* is the predicted grain size based on different values of *n*, and *d* is the actual measured grain size; when the $f_{\mathrm{Error}(n)}$ value is minimized, the predicted grain size obtained from the equation is closest to the actual grain size. The corresponding *n* value is the optimal value.

**Figure 13:**
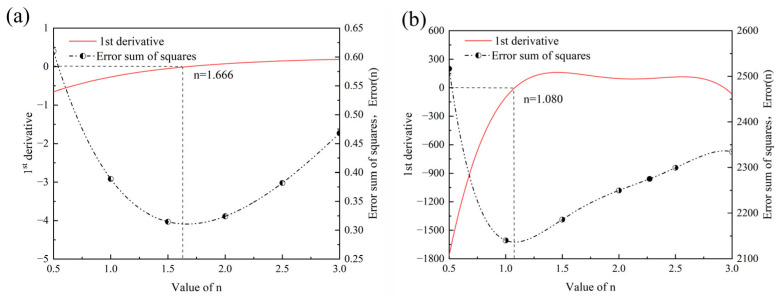
Relationship curves of n and $f_{\mathrm{Error}\left(n\right)}$ at different temperature intervals: (**a**) 900–950 °C and (**b**) 1000–1150 °C.

Figure 13 shows the relationship curves of *n* and $f_{\mathrm{Error}(n)}$ at different temperature intervals, with the corresponding curve equations:

(6) $$f_{\mathrm{Error}\left(n\right)}=1.085-1.302n+0.821n^{2}-0.259n^{3}+0.0468n^{4}-0.00369n^{5}\;(900\sim 950\;{}^{\circ}C)$$

(7) $$f_{\mathrm{Error}\left(n\right)}=4432.029-6605.357n+7181.873n^{2}-3705.565n^{3}+927.899n^{4}-90.650n^{5}\;(1000\sim 1150\;{}^{\circ}C)$$

By taking the derivative of the relationship curve of *n* and $f_{Error\left(n\right)}$, the lowest $f_{Error\left(n\right)}$ value corresponds to the optimal *n* value when the derivative function is 0. At a heating temperature of 900–950 °C, the optimal *n* value is 1.666 (Figure 13a), and at a heating temperature of 1000–1150 °C, the optimal *n* value is 1.08 (Figure 13b). The grain growth model formulas for different temperature intervals can be obtained as follows:

(8) $$d_{t}^{1.666}-d_{0}^{1.666}=853.811\times t^{1.3}\exp\left(-\frac{94251.924}{RT}\right)\;\;\;(900\sim 950\;{}^{\circ}C)$$

(9) $$d_{t}^{1.08}-d_{0}^{1.08}=462217.112\times t^{0.791}\exp\left(-\frac{134390.156}{RT}\right)\;\;\;(1000\sim 1150\;{}^{\circ}C)$$

To further verify the reliability of the grain growth mathematical model, the mean absolute percentage error (*MAPE*) and the coefficient of determination (COD, *R^2^*) are introduced [28,29]. When the value of *MAPE* is small, the established mathematical model fits the actual situation better. Usually, an *MAPE* less than 10% is considered to indicate a better prediction model with high prediction accuracy. The calculation equation is as follows:

(10) $$MAPE=\frac{1}{N}\sum_{i=1}^{N}|\frac{Y_{i}-X_{i}}{X_{i}}|\times 100\%$$

**Figure 14:**
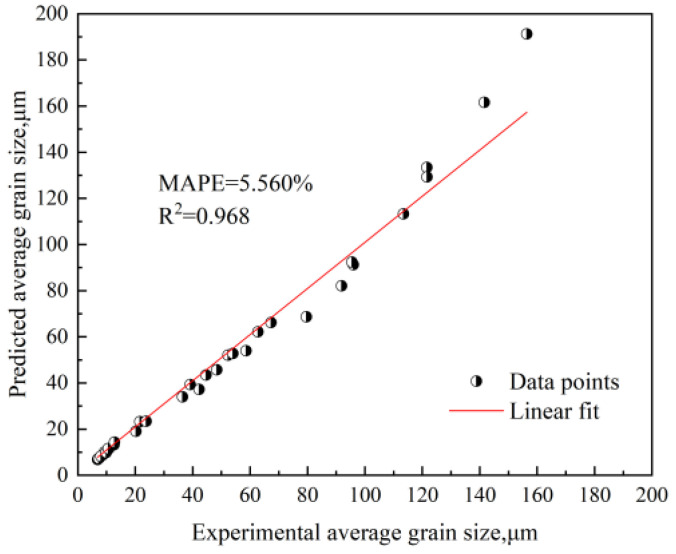
Comparison of actual measured grain size and calculated value.

The COD (*R^2^*) represents the degree to which the model explains the variability in the data. The closer the value of *R^2^* to 1, the better the model fits the test data. Its equation is as follows:

(11) $$R^{2}=1-\frac{\sum_{i=1}^{N}(X_{i}-{Y_{i})}^{2}}{\sum_{i=1}^{N}(X_{i}-\bar{X}{)}^{2}}$$

where *X_i_* and *Y_i_* are the actual measured grain size and the grain size predicted by the equation, respectively; *N* is the total number of experimental samples; and $\bar{X}$ is the average value of the actual measured grain size. Figure 14 provides a comparison chart of the actual measured grain size and the calculated value, where the two can be seen to match well, with an *MAPE* of 5.560% and an *R^2^* = 0.968, indicating that the model can reasonably predict the grain growth of the experimental steel for different temperature intervals.

## 5. Conclusions

The grain growth behavior of steel was studied within the temperature range of 950–1150 °C and at holding times of 0–120 min. The main conclusions obtained are as follows:

(1) Within the temperature range of 950–1000 °C, the grain growth rate is slow, while within the temperature range of 1000–1150 °C, the grain growth rate is relatively fast because, within the former temperature range, a large number of undissolved M_6_C-type carbides are present in the steel. The pinning effect of the carbides hinders grain growth. However, when the temperature exceeds 950 °C, the carbides dissolve extensively, reducing the pinning effect on the grain boundaries, which accelerates the grain growth rate. In the later solidification process design, the temperature selection should be below 950 °C and as low as possible.

(2) By introducing the error equation, the optimal predictive mathematical model was established for grain growth in the experimental steel. The reliability of the model was verified by the mean absolute percentage error (*MAPE*) and the coefficient of determination (COD, *R^2^*), and the prediction was close to the actual results.

The work of this paper provides a theoretical basis for the design of heat treatment processes in actual production, which is conducive to the regulation of grain size in experimental steel. In the future, it is necessary to continue researching the relationship between mechanical properties and grain size in high-strength stainless steel.

## Data Availability

The original contributions presented in this study are included in the article. Further inquiries can be directed to the corresponding authors.
